# Spatial heterogeneity of cell-matrix adhesive forces predicts human glioblastoma migration

**DOI:** 10.1093/noajnl/vdaa081

**Published:** 2020-07-03

**Authors:** Rasha Rezk, Bill Zong Jia, Astrid Wendler, Ivan Dimov, Colin Watts, Athina E Markaki, Kristian Franze, Alexandre J Kabla

**Affiliations:** 1 Department of Engineering, University of Cambridge, Cambridge, UK; 2 Department of Clinical Neuroscience, University of Cambridge, Cambridge, UK; 3 Department of Physiology, Development and Neuroscience, University of Cambridge, Cambridge, UK

**Keywords:** cell-matrix adhesion, cell migration, glioblastoma

## Abstract

**Background:**

Glioblastoma (GBM) is a highly aggressive incurable brain tumor. The main cause of mortality in GBM patients is the invasive rim of cells migrating away from the main tumor mass and invading healthy parts of the brain. Although the motion is driven by forces, our current understanding of the physical factors involved in glioma infiltration remains limited. This study aims to investigate the adhesion properties within and between patients’ tumors on a cellular level and test whether these properties correlate with cell migration.

**Methods:**

Six tissue samples were taken from spatially separated sections during 5-aminolevulinic acid (5-ALA) fluorescence-guided surgery. Navigated biopsy samples were collected from strongly fluorescent tumor cores, a weak fluorescent tumor rim, and nonfluorescent tumor margins. A microfluidics device was built to induce controlled shear forces to detach cells from monolayer cultures. Cells were cultured on low modulus polydimethylsiloxane representative of the stiffness of brain tissue. Cell migration and morphology were then obtained using time-lapse microscopy.

**Results:**

GBM cell populations from different tumor fractions of the same patient exhibited different migratory and adhesive behaviors. These differences were associated with sampling location and amount of 5-ALA fluorescence. Cells derived from weak- and nonfluorescent tumor tissue were smaller, adhered less well, and migrated quicker than cells derived from strongly fluorescent tumor mass.

**Conclusions:**

GBM tumors are biomechanically heterogeneous. Selecting multiple populations and broad location sampling are therefore important to consider for drug testing.

**Key Points**

- GBM tumors are biomechanically heterogeneous.
- GBM cell migration is inversely correlated with cell-matrix adhesion strength.
- 5-ALA fluorescence intensity during surgery correlates with the motility properties of GBM cells.

**Importance of the Study**

This study shows a clear correlation between the mechanical behavior of patient-derived tumor cells and intraoperative tissue fluorescence using 5-aminolevulinic acid (5-ALA). Using a novel microfluidics approach, we measure cell-matrix adhesive forces (within and between tumor samples) and suggest a biophysical relationship between the characteristics of GBM cells and tumor spatial structure. Cells derived from the weak fluorescent tumor rim and nonfluorescent tumor margins have different adhesion profiles and are highly migratory compared to those found in the core of the tumor. Not accounting for internal sampling location within each tumor obscures differences in cell morphology, motility, and adhesion properties between patients. Preclinical tests aimed at developing a treatment for GBM using anti-invasive drugs or adhesion inhibitors would benefit from using cell lines derived from the tumor periphery (with low 5-ALA intensity) rather than cell lines derived from the tumor core.

Glioblastoma (GBM) is the most common and most malignant brain tumor. No cure is available despite significant improvements in surgical techniques and radiation technology. The extensive and infiltrative growth pattern of GBM makes surgical resection extremely difficult^1^ and limits the efficacy of radiation therapy by obscuring tumor margins.^2^ Tumor recurrence is inevitable; targeted chemotherapy^3^ and immune therapy^4^ have failed to stop tumor recurrence.

Migration and invasion are driven by mechanical forces.^5^ The mechanical properties of a GBM tumor and its microenvironment have also shown to contribute to tumor invasion.^6,7^ GBM employs a mesenchymal mode of migration^8^ using focal adhesions proteins as molecular clutches to transmit forces to their environments. Targeting integrins or kinases that mediate cell-matrix adhesion has therefore been explored as a strategy to inhibit tumor growth (eg, focal adhesion kinase [FAK] inhibitor^9^) or to halt the mesenchymal mode of migration employed by infiltrating GBM cells.^10^ However, therapeutics designed to target adhesion receptors or proteases have failed in clinical trials,^11^ particularly in gliomas (eg, cilengitide^12^). This failure might be due to the heterogeneity in expression of the adhesion proteins in glioma. Arguably GBM intratumor heterogeneity is the key to understanding treatment failure^13^ and infiltration.

GBM tumors show transcriptomic and genomic distinct subclasses^14,15^ which vary across patients, but also across individual cells within a tumor.^16^ This could explain why gene expression-based molecular classification of brain tumors failed to provide a more accurate prediction of tumor progression and response to treatment. Differential response to treatment is visible during fluorescent-guided surgery. GBM cells glow fluorescent pink when 5-aminolevulinic acid (5-ALA) is administered orally prior to surgery. The heterogeneity of 5-ALA-induced PpIX fluorescence observed during surgery was associated with different cellular functions and a distinct mRNA expression profile, where nonfluorescent tumor tissue resembled the neural subtype of GBM and fluorescent tumor tissue did not exhibit a known subtype.^17^ However, whether fluorescence heterogeneity is mirrored by physical heterogeneity among GBM cells remains unclear.

To investigate the different adhesive and migratory properties of GBM subpopulations, we derived tumor cells from different GBM patients and from different regions within the same tumor. Tissue samples obtained from the same tumor were collected from spatially distinct locations with different 5-ALA fluorescent intensities. We adapted a microfluidic device for detachment of adherent cells through shear stress.^18,19^ We demonstrate substantial intra- and intertumoral heterogeneity; adhesion strength varied 10-fold (from 15 to 150 Pa). Cells from the weak fluorescent tumor rim and nonfluorescent margins were smaller, less adherent with highly migratory behavior, suggesting that differential adhesion and migration speed between subpopulations of cancer cells may contribute to tumor invasion. Such heterogeneity could also explain recently observed differential responses of patients to adhesion-blocking drugs.

## Materials and Methods

### Sample Collection

**Figure 1.**
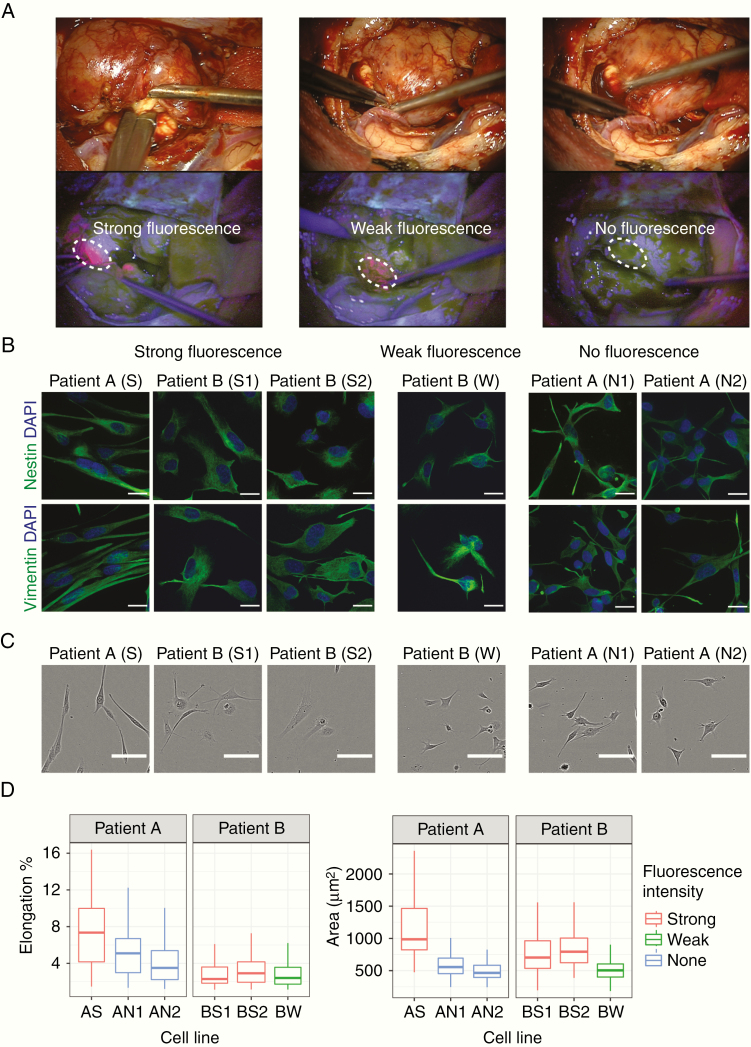
Fluorescence and morphological characteristics of GBM patients derived cell lines. (A) The white light image of the resection cavity and corresponding fluorescence view of the surgical field demonstrating the heterogeneous fluorescence pattern of 5-ALA (bright strong pink from tumor core, faint/weak pink from tumor rim, and nonfluorescent tumor margin). Intraoperative images were obtained via ZEISS OPMI PENTERO 800, with 39× magnification. We collected 3 strongly fluorescent tissue samples from the tumor mass (S from patient A, S1 and S2 from patient B), 2 nonfluorescent tumor margins (N1 and N2) from patient A, and 1 weak fluorescent tissue sample (W) from patient B. (B) Immunofluorescence staining for evidence of GBM stem-like cells. Neural stem cell marker nestin (green) and vimentin (green) are expressed in each cell line; scale bars 20 µm. (C) Representative phase-contrast images showing cells derived from strong fluorescent tissue samples have a larger area compared to cells derived from weak- and nonfluorescent samples. Patient A exhibited a more elongated morphology compared to patient B (***P < .001); scale bars 100 µm. At least 3 independent experiments were carried out, with between 360 and 500 cells were analyzed from at least 3 independent experiments per patient. (D) Morphological quantification of cells cultured on PDMS substrates of approximately 1.5 kPa stiffness. Boxplots show spatial and intertumoral heterogeneity in cell area and cell elongation. Boxplots represent the “minimum,” first quartile (Q1), median, third quartile (Q3), and maximum. Between 110 and 180 cells were analyzed from at least 2 independent experiments.

Tissue collection protocols complied with the UK Human Tissue Act 2004 (HTA license ref. 12315) and have been approved by the local regional ethics committee (LREC ref. 04/Q0108/60). Tissue samples were derived from newly diagnosed GBM patients who underwent their first surgical resection at Addenbrooke’s, Cambridge University Hospitals. 5-ALA fluorescence was orally administered 4 h before induction of anesthesia at a dosage of 20 mg/kg. Three different regions within the tumor were biopsied. Six tissue samples from 2 different patients were taken from spatially separated sections using MRI stealth imaging. Navigated biopsy samples were collected from strongly fluorescent tumor cores, a weak fluorescent tumor rim, and nonfluorescent tumor margins (Figure 1A). In addition, 3 tissue samples from a third patient were collected from similar locations (results described in Supplementary Material). The patient’s clinical information and their molecular biomarker status (IDH mutation and *MGMT* promoter methylation) can be found in Supplementary Table S1.

### Derivation of GBM Stem-Like Cells

Cell derivation and maintenance have previously been described.^20^ Briefly, tissue was mechanically minced and enzymatically dissociated before passing through a 40 µm cell strainer. Cells were seeded in serum-free medium (SFM; phenol red-free Neurobasal A) with 2 mM l-glutamine and 1% volume/volume (v/v) penicillin/streptomycin (PS) solution with 20 ng/mL human epidermal growth factor, 20 ng/mL zebrafish fibroblast growth factor (FGF-2), 1% v/v B27 SF supplement, and 1% N2 SF supplement. Cells were allowed to form primary aggregates. Spheroid aggregates were collected and plated onto Engelbreth-Holm-Swarm sarcoma extracellular matrix (ECM, Sigma)–coated flasks (ECM 1:10 dilution with HBSS) and allowed to form a primary monolayer. When the primary monolayer reached 80% confluency, cells were passaged to generate the subsequent monolayers by mechanically and enzymatically dissociating remaining aggregates. Cells were maintained at 37°C and 5% CO_2_. Experiments were performed using passages 3–9. Cell lines were screened regularly for mycoplasma.

### Immunocytochemistry

Cells were seeded in duplicates into µ-Dish 35 mm (60 000 cells/ibidi dish). After 48 h, cells were washed with sterile phosphate-buffered saline (PBS, Thermo Fisher) before being fixed in 4% paraformaldehyde for 30 min (10 min at 4°C followed by 20 min at room temperature). Cells were washed and then permeabilized for 4 min in 0.2% Triton in PBS. Cells were washed again and incubated in blocking buffer (1% bovine serum albumin, and 2% normal goat serum, in PBS) for 1 h at room temperature before incubating with the primary antibody for 2 h. The following primary antibodies were used: Nestin (Abcam, ab22035, 1:100), Vimentin (Abcam, ab8069, 1:200), NG2 (Abcam, ab83178, 1:200), Ki67 (Abcam, ab16667, 1:200), Vinculin (FAK100, Sigma 1:200), and F-actin (FAK100, Sigma 1:500). Secondary antibodies were applied for 1 h (goat anti-mouse Alexa Fluor 488 preadsorbed, Abcam, 1:750 dilution and goat anti-rabbit Alex Fluor 594 preadsorbed, Abcam, 1:750). Nuclei were stained with DAPI (Roche).

### Immunoblotting

Cell lysis was performed using cOmpleteTM, EDTA-free lysis-M buffer with protease inhibitor (Roche). Protein concentrations were determined using the Pierce BCA kit (Thermo Scientific). Equivalent amounts of protein were electrophoresed on SDS-polyacrylamide gels. The gels were then electroblotted onto polyvinylidene fluoride membranes. After blocking with Odyssey Blocking Buffer (TBS; LI-COR), membranes were incubated with the primary antibody overnight (paxillin, vinculin, FAK, GAPDH [codes need to be looked up at home]). Finally, the relevant protein was visualized by staining with the appropriate IgG H&L secondary antibody coupled to either IRDye 800CW or IRDye 680RD. The antigen of interest was detected using the LI-COR Odyssey CLx Infrared Imaging System. Results were analyzed using ImageStudio.

### Polydimethylsiloxane Substrates for Studying Cellular Motility and Morphology

NuSil GEL-8100 (NuSil) was prepared in a 1:1 ratio of component A and component B and mixed well for 60 s: 1% (w/w) 10:1 (base/crosslinker w/w) Sylgard-184 (VWR) was added to the GEL-8100 and mixed well for 60 s. For cell morphology experiments, approximately 120 mg of the polydimethylsiloxane (PDMS) was added to µ-Dish 35 mm (120 mg/dish). For cell migration experiments, 80 mg per well of PDMS was added to 24-well culture plates (Corning Life Science). Coated vessels were baked at 65°C for 13 h. This treatment gave a shear modulus value of *G* = 1.53 ± 0.12 kPa, *n* = 16, verified by atomic force microscopy (AFM) indentation. The AFM setup consisted of a JPK CellHesion 200 scanner, and the indentation probe was made by gluing a spherical polystyrene particle (90 um diameter) to a tipless AFM probe (SHOCON-TL, *k* ~ 0.1 N/m). Vessels were sterilized by immersion in 70% (v/v) ethanol in distilled water for 15 min, followed by 2 rinses with PBS. The PDMS surface was coated with ECM at a concentration corresponding to a surface density of 6.67 µg/mL assuming complete adsorption. 

### Cell Detachment Assay

Microfluidic devices were manufactured (Supplementary Information S1) and sterilized by immersing in 70% ethanol in distilled water (v/v) in a Petri dish and perfusing with the same liquid at 10 mL/h for 30 min. The devices were lifted from the ethanol and perfused with phosphate-buffered saline (PBS, Thermo Fisher) at 5 mL/h for 1 h to rinse the ethanol and allow PBS to permeate the bulk of the device. The devices were filled with 200 µg/mL ECM matrix and placed in the incubator overnight (approximately 16 h). SFM was equilibrated overnight in the incubator to minimize bubble formation. Cells were detached as described previously and resuspended to a concentration of 3.5 × 10^6^ cells/mL. Cells were loaded into 1 mL syringes and perfused into the devices at 30 µL/h for 5 min. Perfusion was resumed for 3 min with the inlet and outlet reversed to seed cells evenly. The devices were placed in the incubator for 4 h to promote cell attachment. The devices were then perfused for 20 h with equilibrated SFM at a flow rate of 10 µL/h to allow cells to fully spread.^21–23^ For cell detachment, the neurobasal medium without supplements was also equilibrated overnight in the incubator to minimize bubble formation and to control dissolved gas concentration and pH. Cells were subjected to a steady flow rate of 0.16, 0.33, and 0.5 mL/min to create a constant shear force on the cell (Supplementary Information S1). Phase-contrast images of a single field of view were taken every 2 s at 160× magnification (Zeiss Axio Observer.Z1). The maximum flow rate used was 0.5 mL/min (shear stress of 75.96 Pa), although shear stress of up to 506 Pa is achievable with the system, limited by the maximum flow rate of the syringe pump.

### Time-Lapse Measurements and Cell Tracking Analysis

Cells were cultured in 24-well culture plates (Corning Life Science) according to the manufacturer’s instructions and visualized using a real-time cell imaging system (IncuCyte live-cell ESSEN BioScience Inc.,). Ten thousand cells per well were cultured on PDMS substrate of 1.5 kPa stiffness. Cells were seeded in triplicates and imaged every 10 min for 48 h. Time-lapse images were acquired with IncuCyte, a live cell imaging microscope.

Raw images were processed in CellProfiler^24^ to detect cell outlines. Cells were tracked using automated tracking in TrackMate.^25^ Cell area was calculated after 24 h, using the hierarchical K-means thresholding module in Icy.^26^ The mean square displacement (MSD) of each cell from its starting position was calculated. The MSD of actively moving cells should be larger than the expected MSD of a freely diffusing (Brownian) particle of comparable size:

$$\begin{array}{l} MSD>\frac{k_{B}T}{3\;\pi \;\eta \;r}\;t \end{array}$$

where $k_{B}$ is the Boltzmann constant, T = 310 K the absolute temperature, η the viscosity of the medium, r the radius of the cell, and t the time interval. We assumed the viscosity of the medium to be ≈0.78 mPa s.

### Statistical Analysis

Data were verified from at least 2 independent biological experiments. For experiments involving single-cell analysis, *n* ≥ 100 cells. The order of data collection was randomized; no blinding was performed and no data were excluded from the analysis. 

Morphological differences between patients and fluorescence groups were tested using linear regression. First, we tested differences between patients and differences between fluorescence groups independently. Then we tested for differences simultaneously using an additive model (for patients and fluorescence intensity). Since there was no statistically significant difference between weak- and nonfluorescent cell lines but both were different from strongly fluorescent cell lines, we pooled the weak- and nonfluorescent groups. To check whether sampling from multiple locations violated our standard linear model assumptions, we tested unexplained heterogeneity using mixed-effect regression. Including a random effect of all lines did not significantly improve model fit. This indicates that a mixed effect model is not required.

Adhesion heterogeneity between cell lines was analyzed using Welch’s *t*-test (unequal variance) with Bonferroni correction (15 comparisons). The shear stress required for 50% cell detachment was found by performing an orthogonal distance regression, and the resulting fitted values and associated standard errors were used in the Welch’s *t*-test formula. Differences in cell migration between patients and fluorescence groups were tested using weighted linear regression (using MSD slope estimate as the outcome). First, we tested differences between patients and differences between fluorescence groups independently. After pooling the weak- and nonfluorescent cell lines, we tested for differences simultaneously using an additive model (for patients and fluorescence intensity).

Statistical analyses and plotting were performed in Python 3.6, MATLAB, or R statistical software package.

## Results

### Relationship Between Tissue Fluorescence Heterogeneity and GBM Cell Morphology

Primary GBM cell lines maintained in serum-free media expressed neural stem cell markers nestin and vimentin (Figure 1B). This confirmed the presence of glioma stem-like cells which are regarded as tumorigenic and associated with the heterogeneity in GBM.^27^ We then investigated the cell spreading area which is known to be an indicator of how cells mechanically interact with their extracellular environment.^28^

Cells were cultured on PDMS, with a low modulus consistent with the stiffness of the environment that GBM infiltrate, which ranges from 0.1 to 10 kPa.^29^ Glioma cells are known to spread on substrates of stiffness around 1 kPa.^30^ However, on an extremely compliant substrate (~150 Pa) GBM cells exhibit rounded morphologies with diffuse distributions of F-actin that are unable to migrate productively.^31^ To compare between lines, we used a single stiffness, approximately 1.5 kPa (Methods; Figure 1C).

To explain the variability in cell morphology observed among patients’ cell lines (Figure 1D), we first tested whether cells from patient A differed from those of patient B. Cells derived from patient A exhibited a more elongated morphology compared to cells derived from patient B (***P < .001), but there was no significant difference in cell area (*P* = .1). However, cells derived from strongly fluorescent lines differed significantly in cell area and cell elongation between patients (***P < .001). We therefore adjusted our model to account for fluorescence intensity when comparing between patients (Methods). We found that cells from patient A were both larger and more elongated than cells derived from patient B (***P < .001).

The results demonstrate morphological differences within each tumor and between tumors and show that this heterogeneity is related to 5-ALA fluorescence intensity. Not accounting for this variability obscured the difference between patients. To investigate whether the differences in cell morphology relate to the way cells adhere to their environment, we explored whether heterogeneity in cell area and shape correlate with specific patterns of key cytoskeletal proteins.

### The Organization of Actin Filaments and Vinculin Suggests Intertumoral Heterogeneity in GBM Cell-Matrix Adhesion

**Figure 2.**
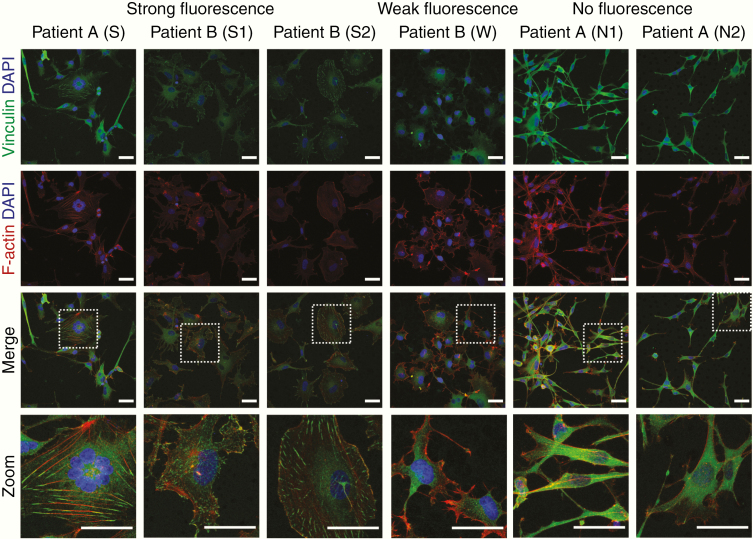
F-actin cytoskeleton and vinculin proteins expression in GBM lines. Confocal images showing the colocalization of vinculin and F-actin at the edge of cells derived from strong fluorescent lines compared to cells derived from weak- and nonfluorescent lines. Cells were stained with TRITC-phalloidin (red) to visualize the actin cytoskeleton, vinculin (green), and DAPI (blue). Scale bars 40 µm.

Actin filament disassembly is required for cell spreading, and the binding to proteins such as paxillin and vinculin is necessary for focal adhesion development.^32^ We imaged the localization and structure of actin filaments and the actin-binding protein, vinculin. The shape and distribution of actin and vinculin differed between cells derived from strongly fluorescent lines and cells derived from weak- and nonfluorescent lines. The colocalization of vinculin with F-actin was evident at the edge of cells derived from strongly fluorescent lines (Figure 2A). Adhesion proteins paxillin, vinculin, and FAK were expressed across all cell lines (Supplementary Figure S1), but their expression levels did not reflect the arrangements and assemblies revealed in immunocytochemical images. The binding of vinculin to F-actin suggests spatial differences in focal adhesion assembly and enlargement within the tumor. We therefore built a setup to quantify the intra- and intertumoral heterogeneity in cell-matrix adhesion.

### Cell-Matrix Adhesion Heterogeneity Within and Between Patient-Specific Tumor Samples

**Figure 3.**
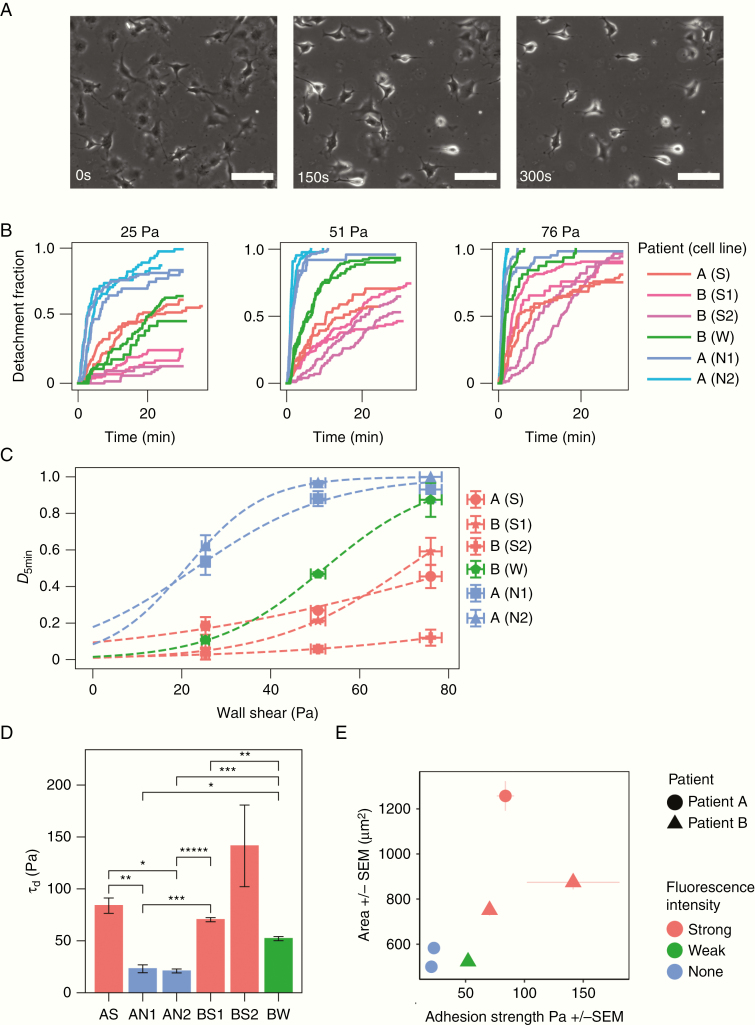
GBM exhibits spatial and intertumor heterogeneity in cell-matrix adhesion. (A) Representative phase-contrast images of cells under microfluidic shear detachment at 0, 150, and 300 s. Here, BW cells are exposed to 0.33 mL/min of flow, corresponding to 51 Pa of shear stress. Scale bars 100 µm. (B) For each condition (shear flow), the detachment–time curve was generated using 2 independent experiments per cell line (25–138 cells per experiment). Detachment increases with shear stress, but the relative ordering of the lines remains constant except for cell line AS. Detachment initially occurs rapidly and plateaus for most cell lines. (C) The inflection point of each sigmoidal curve was extracted to define *τ*_d_, a measurement for the cell-matrix adhesion strength of the cell lines. (D) Differences between weak- and strongly fluorescent lines were statistically significant for all intrapatient pairs except BW and BS2 (single asterisk indicates *P < .05 and additional asterisks indicate further orders of magnitude, under Welch’s *t*-test with Bonferroni correction). The mean value of *τ*_d_ for patient B was significantly higher than that of patient B (**P = .005). (E) Relationship between cell spread area and cell-matrix adhesion strength. Strong fluorescent cells are more adherent and larger than weak- and nonfluorescent cells. Error bars represent SEM from at least 2 independent experiments.

To quantify adhesion strength within the GBM cell population, we built a microfluidic device that supported the overnight culture of GBM cells and generated sufficient shear force to trigger cells detachment (Supplementary Figure S2). Patients’ derived cells were delivered into the channels via syringe pumps (3.5 × 10^6^ cells/mL). Cells were gently perfused with SFM and placed in an incubator for 24 h to allow adhesion to fully establish and cells to fully spread.^21–23^ Cells were subjected to a controlled flow rate to create a constant shear force on the cell (Supplementary Information S1). The fraction of detached cells was measured over time (Figure 3A). We consistently found an initial phase of rapid detachment followed by the second phase of slower detachment (Figure 3B), consistent with previous parallel-plate and microfluidic detachment assays.^33,34^ This transition manifests itself in most experiments as a “knee” in the time–detachment curve after the first 5–10 min of exposure to the shear force (Supplementary Figure S3).

There is some evidence suggesting that the second phase is a result of adhesion weakening in response to imposed flow, driven by Rho regulation of focal contact maturation and turnover.^35,36^ As a consequence, cell detachment measurements taken later in time may not be representative of cell-matrix adhesion under normal conditions. We chose to take measurements of detachment at 5 min of flow, as quantification of adhesion strength (Supplementary Figure S3).

Detachment at 5 min was expressed as a fraction of the cell number at *t* = 0. Detachment fraction as a function shear stress was fitted to a logistic function, and the shear stress required for 50% detachment ($\tau_{d}$) was identified as the inflection point of the logistic fit (Figure 3C). $\tau_{d}$ ranged between 15 and 141 Pa. This range is consistent with microfluidic single-cell adhesion strength measurements of 3T3 fibroblasts, which fell between 20 and 220 Pa.^19^

Cells derived from weak- and nonfluorescent cell lines had significantly lower cell-matrix adhesion than cells derived from strongly fluorescent cell lines across patients A and B (*P < .05; Figure 3D) and smaller cell spreading area (Figure 3E). The mean value of detachment strength of patient B was significantly higher than that of patient A (**P = .005). The data show that both intra- and intertumoral cell-matrix adhesion heterogeneity is present within GBM.

### Migratory Behavior of Tumor-Derived Cell Populations is Predicted by Adhesive Forces

To measure the migratory behavior of GBM cells, we recorded their trajectory on a compliant PDMS substrate. Their movement is stochastic at the scale of 10 min and is best characterized by an effective diffusion coefficient *D* which captures the rate of change of the squared end-to-end distance traveled (MSD Δ*R*^2^) as a function of time interval Δ*T*: Δ*R*^2^ = *D* Δ*T.* The larger *D*, the more migratory the cell line is.

**Figure 4.**
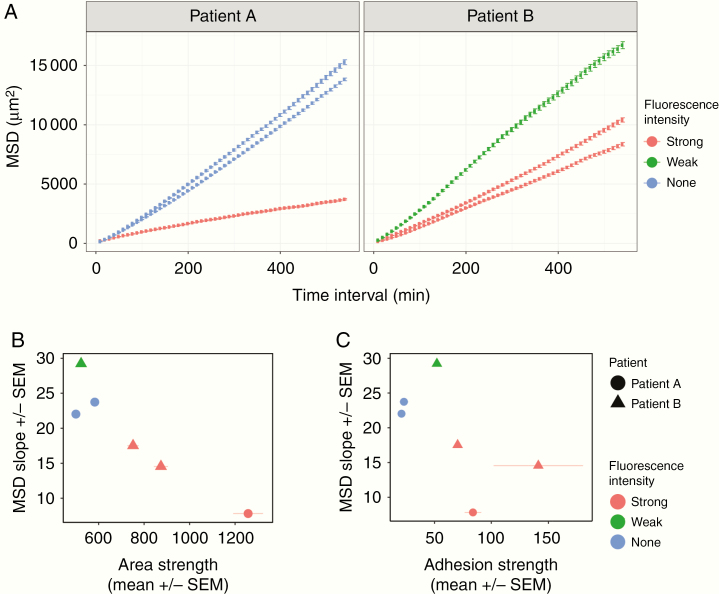
Cell-matrix adhesion is an important factor for GBM cell migration. (A) MSD as a function of time interval (Δ*t*) calculated from cell trajectories with vertical bars representing standard errors. At least 100 cells were analyzed from 2 independent experiments. Having accounted for tissue fluorescence intensity, we found that cells from patient B migrated faster than patient A (*P < .05). Cells derived from strongly fluorescent tissue samples were significantly slower than cells derived from weak- and nonfluorescent cell lines (**P < .01). (B) Cells with larger cell area migrate less than those with smaller cell size derived from the weak- and nonfluorescent samples. Error bars represent SEM from at least 2 independent experiments. (C) Cells derived from strong fluorescent tissue samples are more adhesive and less migratory. Error bars represent SEM from at least 2 independent experiments.

We tested for differences in diffusion coefficient *D* using an additive model (to account for patients and fluorescence intensities), see Methods, and found that cells from patient B migrated faster than patient A (*P < .05). Cells derived from strongly fluorescent tissue samples were significantly slower than cells derived from weak- and nonfluorescent cell lines (**P < .01; Figures 4A), had a larger area (Figure 4B), and higher adhesion strength (Figure 4C). This result is consistent with previous models of pseudopodial migration, in which a certain level of adhesion is required for the generation of traction forces, but excessive adhesion impedes motion.^37^ Additionally this observation suggested a potential mechanism for differential diffusion.

## Discussion

In order to invade the surrounding tissue, GBM cells have to exert forces on their environment and mechanically interact with it. They also have to undergo biological and morphological changes that allow them to migrate through the perivascular spaces and white matter tracts of the brain.^10^ Instead of observing the tumor tissue in vivo, our approach isolated cell lines (from different tumor regions with different intraoperative 5-ALA-induced fluorescence intensities) and measured their adhesive and migratory properties under controlled conditions allowing direct comparison. The results are therefore not confounded by any spatial differences in ECM composition, nutrient, and oxygen availability.

Our findings establish that patient-derived tumor cells have distinct adhesion profiles that were reproducible and consistent over several passages (Figure 3B). These profiles were strongly associated with different migration behaviors, cell size (rather than shape, Supplementary Figure S4), tissue sampling locations, and strong versus weak 5-ALA fluorescence. The relationship was further confirmed by analyzing a third patient whose tissue samples were obtained from 3 similar locations in the tumor; the derived cell lines exhibited similar morphology (Supplementary Figure S5), adhesive (Supplementary Figure S6) and migratory behavior (Supplementary Figure S7). The migratory and adhesion properties of the cell lines were stable over several passages (Figures 3B and 4A; Supplementary Figures 6A and 7A) for the same microenvironment. This suggests that the observed biomechanical heterogeneity reflects specific cell populations rather than cellular plasticity as seen for the expression of various cell surface markers commonly used to define stem cell populations.^27,38^ However, in order to validate this hypothesis, both cell lines and original tumor samples should be genetically tested and analyzed for differences in their mutational and copy number landscape.

The fact that adhesion strength is associated with reduced movement^39^ in vitro suggests a simple model where migratory forces must overcome adhesion forces to generate motion. This idea was tested numerically (Supplementary Information S2) in order to relate MSD curves with the measured values of cell adhesion. This approach can be extended to simulate mixed populations of core and marginal cells. The variation in adhesion strength leads to variations in the amount of migration, causing cells with lower adhesion to be predominant at the leading edge, consistent with the clinical observation (Supplementary Figure S8). This suggests that differential migration may play a role in the spatial distribution of cells within the tumor. Differential migration is known to contribute to cell segregation.^40^ GBM intratumoral cell proliferation is strongly associated with the fluorescence intensity of samples and hence the spatial density and distribution of cells.

Recently, it has also been shown that fluorescent and nonfluorescent tissue samples can be distinguished genetically.^17^ Due to our controlled experimental conditions, much of the observed intra- and intertumoral heterogeneity is likely to be of genetic or epigenetic origin, rather than caused by spatial differences in the original tissue’s microenvironment. A relationship between glioma mechanics, genetic profiles and epithelial–mesenchymal transition marker expression and intraoperative techniques (eg, 5-ALA intensities, stealth imaging) would provide a greater understanding of why specific tumor subtypes are more or less sensitive to current therapeutics.

Our results are consistent with a broader picture of GBM as genetically heterogeneous cancer.^13,16^ The human genome encodes 24 different integrin subunits,^41^ which are highly heterogeneously expressed within GBM.^42–44^ These integrins have varying degrees of cross-specificity in both the natural ECM ligands and synthetic inhibitor molecules they can bind. Our results and previous experimental^45^ and theoretical work^37^ support the hypothesis that the migratory phenotype is linked with cell-adhesive forces. Inhibition of one or a few integrins may not significantly change cell-adhesive forces, due to the aforementioned redundancy or compensation through feedback loops that may themselves be driven by cellular mechanosensing.^46^ Integrin inhibitors for glioma therapy have been assessed primarily by their affinity and specificity for the target molecule and their ability to arrest tumor growth.^47^ Our approach of directly measuring adhesive forces provides an overall quantitative assessment of the strength of cell adhesion that accounts for the presence of integrins and enables a direct correlation with migration data.

Patients’ differential response to adhesion inhibitors and anti-invasive molecular treatments may be due to intrinsic differences in GBM cell motility, adhesion, and traction forces. Preclinical tests of adhesion-block typically use only 1 or 2 tumor core cell lines, which may not be representative of the distribution of integrin expression and adhesion in GBM tumors. Given that integrins play a crucial role in brain tumor infiltration^48^ and GBM intratumor variability in integrin expressions.^49^ New trials that aim to halt GBM recurrence by inhibiting radiation-induced invasion gains and signaling changes,^10,50^ or kinase inhibitors,^9^ could benefit from accounting for intra- and intertumoral differences in cell migration.

## Supplementary Material

vdaa081_suppl_Supplementary_Material

## Notes

**Authorship statement.** R.R. conceptualized and designed the study. R.R., B.Z.J., A.W., A.E.M., C.W., K.F., and A.J.K. designed and performed the experiments, derived the models, and analyzed the data. I.D. performed and analyzed the PDMS compound. C.W. supervised the clinical procedure, provided surgical samples, and interpreted clinical data. All authors discussed the data and wrote the manuscript.
